# scFSNN: a feature selection method based on neural network for single-cell RNA-seq data

**DOI:** 10.1186/s12864-024-10160-1

**Published:** 2024-03-08

**Authors:** Minjiao Peng, Baoqin Lin, Jun Zhang, Yan Zhou, Bingqing Lin

**Affiliations:** 1 School of Mathematical Sciences, Shenzhen University, Nanshan, Shenzhen, 518060 Guangdong China; 2 School of Mathematics and Statistics and KLAS, Northeast Normal University, Renmin Street, Changchun, 130000 Jilin China; 3 Experimental Center, The First Affiliated Hospital of Guangzhou University of Chinese Medicine, Guangzhou, Guangdong 510405 China

**Keywords:** Feature selection, Deep neural network, FDR control

## Abstract

While single-cell RNA sequencing (scRNA-seq) allows researchers to analyze gene expression in individual cells, its unique characteristics like over-dispersion, zero-inflation, high gene-gene correlation, and large data volume with many features pose challenges for most existing feature selection methods. In this paper, we present a feature selection method based on neural network (scFSNN) to solve classification problem for the scRNA-seq data. scFSNN is an embedded method that can automatically select features (genes) during model training, control the false discovery rate of selected features and adaptively determine the number of features to be eliminated. Extensive simulation and real data studies demonstrate its excellent feature selection ability and predictive performance.

**Supplementary Information:**

The online version contains supplementary material available at 10.1186/s12864-024-10160-1.

## Introduction

Single-cell RNA sequencing (scRNA-seq) can reveal heterogeneity and diversity across tissues, organs, and systems at single cell level and has helped researchers improve their understanding of complex biological questions [1, 2]. However, the analysis of scRNA-seq data is challenging. First, scRNA-seq data are over-dispersion. The heterogeneity of gene expression levels in a cell population results in higher variability for scRNA-seq data compared to bulk RNA-seq data [3]. Second, scRNA-seq data are zero-inflated, i.e., excess zeros are observed in typical scRNA-seq data. There are two types of zeros in scRNA-seq data: biological zeros (due to the high heterogeneity between cells, expression levels of some genes are genuinely zero in some cells) and technical zeros (referred to as dropout, some transcripts are missed during the RNA-seq procedure, such as reverse transcription or cDNA amplification steps). Third, features (genes) in scRNA-seq data may be highly correlated [4]. Fourth, with the rapid development of high-throughput sequencing technologies, the sample size of scRNA-seq data increases dramatically [5, 6]. Fifth, scRNA-seq data contains a vast number of features, each representing a gene in the sample cell. As is well-known, the human genome comprises approximately 30,000 genes, and there is typically a small subset of features that genuinely correlates with the response. Together, these characteristics make classification a particularly challenging task for scRNA-seq data.

Currently, there is a large number of approaches that can possibly be applied to classify cells from different conditions in scRNA-seq data. For example, generic classifiers, such as support vector machines and random forest, are potential candidates [7, 8]. And there are some approaches that are specifically designed for RNA-seq data. These approaches mainly rely on the assumption that expression level of each gene follows the zero-inflated negative binomial (ZINB) distribution. The Poisson, the zero-inflated Poisson (ZIP), and the negative binomial (NB) distributions are three special cases of the ZINB distribution. For example, PLDA assumes Poisson distribution for the discrete count data of RNA-seq data [9] and ZIPLDA uses ZIP distribution for RNA-seq data with excess zeros [10]. These two methods apply different techniques to deal with the different aspects of challenges of RNA-seq data. Specifically, PLDA addresses the challenge of over-dispersion through a power transformation, while ZIPLDA models the gene count with ZIP to consider the excess zeros in RNA-seq data and uses the ratio of the sum of squares between groups to that of within groups to select the genes to reduce the dimension. SINC [7], on the other hand, performs classification based on deep neural network. To reduce the dimension of data, SINC conducts an F-test on each gene to test whether means of different classes are significantly different and selects top 1500 genes after ranking the p-values in increasing order.

Deep neural network (DNN) is a highly flexible machine-learning technique and has demonstrated superior performance in various scientific problems. Since DNN enables the capturing of complexity and nonlinearity in scRNA-seq data and is highly scalable, it has the potential to overcome the first four challenges of scRNA-seq data, namely, over-dispersion, zero-inflation, high gene-gene correlation and large sample size [7, 11, 12]. To further boost predictive accuracy and interpretability, employing feature selection within a DNN framework is crucial. Although there are thousands of genes in scRNA-seq data, most of genes are irrelevant to the output and useful information is concentrated in a small number of genes. The main goal of feature selection is to find a subset of the input features that explains the output well. This not only reduces computational resources, but also reduces noise and improves the model generalization on unseen data [13]. Feature selection can also reduce experimental costs since researchers can collect the expression levels of small set of features when making prediction [14]. Additionally, feature selection can enhance interpretability by selecting a subset of features with significant predictive power on the output [11].

The feature selection methods are usually classified into three categories: filter, wrapper and embedded methods [15]. Filter methods select features based on certain criteria which measures the relevance between the features and output, and the selection procedure does not involve the model training. This makes filter methods overlook the interactions among features. On the other hand, wrapper and embedded methods attempt to select features that optimize the performance of a specific learning algorithm. Specifically, wrapper methods evaluate subsets of features based on learning algorithms’ predictive power, while embedded methods select features during the training of the learning algorithm. One type of widely used embedded method involves the regularization of parameters of learning algorithms [16, 17]. For example, spare group Lasso is used to penalize the set of outgoing weights from the same input node in neural networks to impose group-level sparsity on the network’s connections [18, 19]. Another line of research, which is relevant, uses backward elimination procedure to eliminate one or several least irrelevant features among all remaining features. For example, SurvNet, based on newly proposed measure of feature importance and an elimination procedure with FDR control, can adaptively eliminate features and estimate the false discovery rate at each step [11].

In this paper, we propose a feature selection method based on framework of deep neural network for scRNA-seq data. Our method is an embedded method that selects features during model training. The procedure starts with all input features, and sequentially deletes features that have least impact on the fit. Features with the smallest importance scores are candidates for removal. At each step, false discovery rate is estimated to control the quality of remaining features. Due to the inherent advantages of DNN, scFSNN does not impose an assumption of specific statistical distributions for gene expression levels and enables the capturing of the complexity and non-linearity in scRNA-seq data. Furthermore, the implementation of scFSNN procedure that is based on popular deep learning framework, PyTorch, is highly scalable and can be applied to large data sets.

## Methods

Let $\varvec{X}^{'}=x^{'}_{ij}$ denote an $n\times p$ count matrix of scRNA-seq data with *n* cells and *p* genes, where $x^{'}_{ij}$ is the expression level for gene *j* in cell *i*, $i=1,\ldots ,n$ and $j=1,\ldots ,p$. Let $y_i$ denote the output, $y_i$ can be one-dimensional or multi-dimensional. The tuple $(\varvec{x}^{'}_{i.}, y_i)$ represents the *i*th sample. We first normalize $\varvec{X}^{'}$ with total counts for each cell. To train the neural network more efficiently and stably, we also take the logarithm and standardize the normalized data. Let $\varvec{X}=(x_{ij})_{i=1,\ldots ,n; j=1,\ldots ,p}$ be the normalized, log-transformed and standardized data, that is,

1 $$\begin{aligned} x_{ij}= \frac{\textrm{log}\left( x^{'}_{ij}d_0/d_i+1\right) -m_j}{s_j}, \end{aligned}$$

where $d_0$ is the median of total numbers of counts, $d_i$ is the total counts of *i*th cell, $i=1,\ldots , n$, $m_j$ and $s_j$ are the mean and standard deviation of each gene for normalized and log-transformed data.

**Fig. 1:**
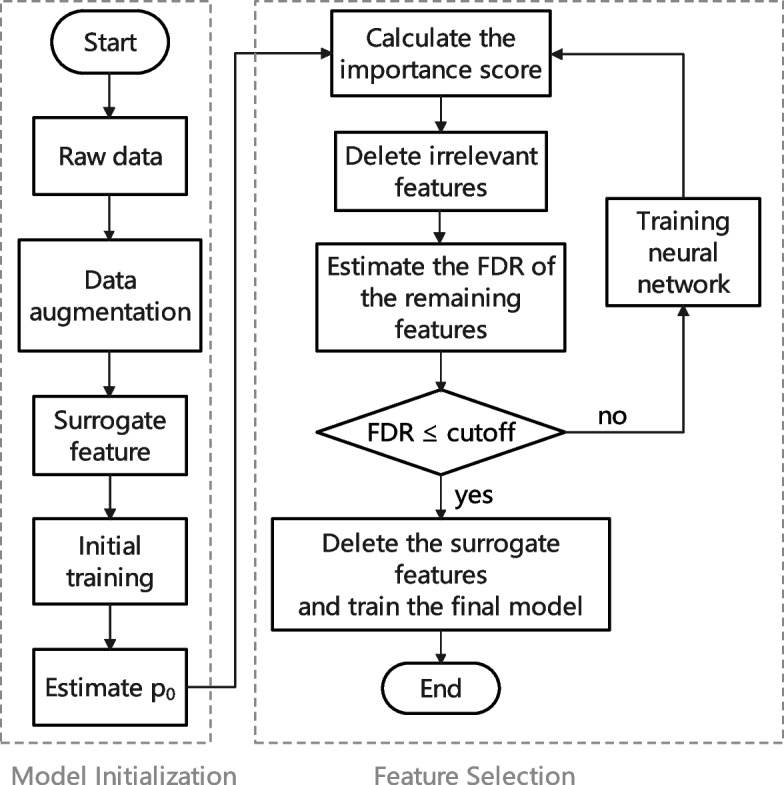
The flow chart of scFSNN. The scFSNN model consists of two parts: model initialization and feature selection. The model initialization process begins with data augmentation and generating surrogate features. It then initializes the model to obtain the estimated value of $p_0$. The second part is the feature selection process. This process initially calculates the importance scores of features and eliminates one or some least important features based on the importance scores. Subsequently, it estimates the False Discovery Rate (FDR) of the remaining features. If the estimated FDR is greater than the given cutoff, the feature selection process continues; otherwise, the feature selection process stops, and the remaining original variables are used to train the final model

An overview of scFSNN is shown in Fig. 1. The architecture of deep neural network of scFSNN is given below:

$$\begin{aligned} B{} & {} = \textrm{ReLU}(XW_{XB})\\ E{} & {} = \textrm{ReLU}(BW_{BE})\\ O{} & {} =\textrm{Softmax}(EW_{EO}),\\ \end{aligned}$$

where *B*, *E* and *O* represent the first hidden, second hidden and output layers. The two hidden layers have 256 and 128 nodes, and all layers are fully connected. Additionally, we use batch normalization [20] and dropout method [21] on each hidden layer, with a dropout rate set to 0.5. The loss function *L* is cross entropy, and an Adam optimizer with a learning rate 0.001 is used for training the model. The batch size is set to 32.

To select truly relevant features with the output, scFSNN starts with all input features and deletes features that have the smallest importance scores at each step. Additionally, we introduce a number of surrogate features known to be irrelevant to the output to estimate the false discovery rate. Similar to SurvNet [11], we define the importance score of *j*th feature as the average of absolute values of $\frac{\partial L(y_i, O_i)}{\partial x_{ij}}$

2 $$\begin{aligned} S_j = \frac{1}{n}\sum \limits _{i=1}^n \left|\frac{\partial L(y_i, O_i)}{\partial x_{ij}}\right|, \end{aligned}$$

where $O_i$ is the output of the network for the *i*th sample. The term $\frac{\partial L(y_i, O_i)}{\partial x_{ij}}$ describes how the loss changes with *j*th feature in the *i*th sample, thus $S_j$ measures the average of loss changes with respect to *j*th feature. Therefore, a larger $S_j$ indicates a larger impact of *j*th feature on the loss function.

In order to determine when we should stop the feature selection procedure and how many features to discard at each step, we need to estimate false discovery rate (FDR) after each training step. Assume that in the original data there are *p* features among which there are $p_0$ irrelevant (null) ones. We introduce *q* known null features to the original data by random sampling from the original data matrix without replacement. Therefore, scFSNN starts with $p+q$ features, at each step, it deletes a number of features with the smallest important scores, which may be original features or surrogate features. Suppose, after several training steps, there are *r* features retained in the neural network, among which there are $r_0$ surrogate features. Thus, the proportion of surrogate features that have not been deleted is $r_0/q$. If we assume the distributions of importance scores for null features from original data and the surrogate features are similar, then the number of null features from original data that still retain in the network is $\frac{r_0}{q}\times p_0$. The estimate of FDR of $r-r_0$ original features is given by

3 $$\begin{aligned} \frac{\frac{r_0}{q}\times p_0}{r-r_0}. \end{aligned}$$

In practice, $p_0$ is usually unknown, and in order to estimate FDR, we need to first estimate $p_0$.

In scFSNN, we initially train the neural network with all features for 30 epochs, and $p_0$ is estimated as

4 $$\begin{aligned} \hat{p}_0 = \textrm{min}(\#\{S_j<S_m\}\times 2, p), \end{aligned}$$

where $S_m$ is the median importance score of surrogate features. If we assume the distributions of importance scores for null features from original data and the surrogate features are similar, the features from original data with importance scores less than $S_m$ are most likely to be null ones and the number of null features from original data is twice of $\#\{S_j<S_m\}$.

Finally, we can estimate the FDR as

5 $$\begin{aligned} \hat{\eta } = \frac{\frac{r_0}{q} \times \hat{p}_0}{r-r_0}=\frac{r_0}{r-r_0} \times \frac{\hat{p}_0}{q}. \end{aligned}$$

In practice, it is also a common strategy to replace $p_0$ by *p* [11, 22]. However, this strategy is too conservative if $p_0$ is much less than *p*, which may be a common situation for scRNA-seq data. Our estimate of $p_0$ can make the estimate of FDR more accurate.

In order to improve the efficiency of the procedure, we delete $\left\lceil \varepsilon \times \left(1-\frac{\eta ^{*}}{\hat{\eta }} \right) \times r_0 \right\rceil$ features each time as [11], where $\varepsilon$ is a constant between 0 and 1, $\eta ^{*}$ is a user-defined threshold. In all experiments in this paper, we set $q=\left\lfloor \frac{p}{2}\right\rfloor$, $\eta ^{*}=0.1$ and $\varepsilon =0.1$. In numerical experiments, we randomly divided the cells in each dataset without replacement into three sets. The first set, containing $64\%$ of the cells, was used for training the deep learning model. The second set, comprising $16\%$ of the cells, served as the validation set for calculating the importance scores and subsequently estimating the FDR. The remaining $20\%$ of cells formed the test set, untouched until the feature selection procedure and parameter estimation were completed.

Though the sample size of scRNA-seq data is often high, certain situations present limitations in obtaining sufficient samples due to factors like limited bioresources, prohibitive costs, or ethical considerations [23]. To further improve predictive performance in small datasets, we augment data based on convex pseudodata (CPD) [24, 25]. Typically, a prediction algorithm performs better if given a large enough sample from the underlying distribution. Data augmentation, which artificially generates additional data from the existing data is a commonly used technique in DNN, especially in computer vision. CPD is a relatively simple and nonparametric data augmentation method and depends only on a single parameter, $d, 0<d<1$. The additional sample is generated in steps as follows.

1. Randomly select two samples $(\varvec{x}_{i.}, y_i)$ and $(\varvec{x}_{j.}, y_j)$ from the original data.
2. Select a random number $\alpha$ from a uniform distribution on the interval (0, *d*).
3. The new generated sample is $(x^{*}, y^{*})$, where $x^{*}=(1-\alpha )\varvec{x}_{i.}+\alpha \varvec{x}_{j.}$ and $y^{*}=y_i$.

In the preprocessing stage, we use the CPD procedure to randomly generate new data $\varvec{X}^{*}$ and use samples from both $\varvec{X}$ and $\varvec{X}^{*}$ to train the model. In all experiments, we set $d=0.2$.

## Results

### Simulation evaluation of scFSNN

To evaluate the feature selection performance of scFSNN in classification analysis of scRNA-seq, we designed the following simulations under extensive settings approximating different biological scenarios. Specifically, we applied the R package Splatter [26] to simulate scRNA-seq read count data. We simulated datasets with two classes, each dataset contains 10000 genes. Here, we consider two studies. In Study 1, we fix the proportion of DE genes as 0.05, and vary the number of cells from 1000 to 5000. In Study 2, we fix the number of cells as 2000, and vary the proportion of DE genes from 0.02 to 0.3. We compared scFSNN with five other classifiers, including SurvNet and four variants of penalized methods. Four variants of penalized methods penalize first hidden layers’ parameters of neural networks by $\textrm{L}_1$, $\textrm{L}_2$, $\textrm{GL}$ (group Lasso) and $\textrm{SGL}$ (sparse group Lasso) [18]. We ran the penalized methods by setting $\lambda$ in the exponential range $10^{-j}$, with *j* going from 1 to 5 on eight real datasets (Supplementary Figs. S1, S2). We can see that from $10^{-3}$ onwards, their accuracies are basically indistinguishable as shown in [18]. Hence, in all numerical experiments, we report the results for penalized methods with fixed tuning parameter $10^{-3.5}$ to reduce the computational complexity.

Since the predictive accuracy of all methods is close to 1 in these relatively simple binary classification settings, here we mainly use FDR to evaluate the feature selection ability of scFSNN. We repeat the simulation 20 times for each setting. Study 1 examines the effect of sample sizes on the classification. It is shown that the FDRs of scFSNN and SurvNet are less than the prespecified threshold in all settings with different sample sizes, and the FDRs of $\textrm{L}_1$ and SGL decrease with an increasing number of sample sizes (Table 1). This indicates that scFSNN and SurvNet perform significantly better than penalized methods, even when the sample size is small. Study 2 explores the effect of the number of differentially expressed genes on the binary classification. It is shown that the FDR of all methods decreases with the increasing number of differentially expressed genes (Table 2). scFSNN also demonstrates its superiority over the other methods in Study 2.

**Table 1:** FDRs of scFSNN and five other classifiers on five simulated scRNA-seq datasets with different sample sizes. False Discovery Rate (FDR) represents the proportion of features identified as statistically significant but actually irrelevant to the response, among all discovered features. Here, we report the average FDR across 20 replicate experiments. Standard errors are shown in parentheses

Sample size	scFSNN	SurvNet	$\textrm{L}_1$	$\textrm{L}_2$	GL	SGL
1000	0.0437	0.0757	0.6897	0.9505	0.4619	0.4816
	(0.0300)	(0.0205)	(0.2789)	(0.0030)	(0.4238)	(0.4527)
2000	0.0359	0.0775	0.6357	0.9505	0.5329	0.2795
	(0.0201)	(0.0336)	(0.3315)	(0.0032)	(0.4261)	(0.4181)
3000	0.0381	0.0620	0.6328	0.9505	0.5817	0.3005
	(0.0277)	(0.0291)	(0.3207)	(0.0031)	(0.3898)	(0.4175)
4000	0.0451	0.0748	0.6671	0.9505	0.5093	0.2617
	(0.0290)	(0.0317)	(0.2503)	(0.0031)	(0.4179)	(0.3898)
5000	0.0334	0.0686	0.6611	0.9505	0.4942	0.3759
	(0.0299)	(0.0307)	(0.3265)	(0.0031)	(0.4417)	(0.4064)

**Table 2:** FDRs of scFSNN and five other classifiers on six simulated scRNA-seq datasets with different proportions of DE genes. False Discovery Rate (FDR) represents the proportion of features identified as statistically significant but actually irrelevant to the response, among all discovered features. Here, we report the average FDR across 20 replicate experiments. Standard errors are shown in parentheses

DE	scFSNN	SurvNet	$\textrm{L}_1$	$\textrm{L}_2$	GL	SGL
0.02	0.1281	0.0901	0.8962	0.9800	0.5579	0.5137
	(0.0753)	(0.0523)	(0.1432)	(0.0015)	(0.4372)	(0.3881)
0.03	0.0537	0.0733	0.7996	0.9698	0.3956	0.3358
	(0.0394)	(0.0381)	(0.2211)	(0.0019)	(0.4145)	(0.4077)
0.05	0.0368	0.0727	0.6859	0.9506	0.3738	0.1879
	(0.0157)	(0.0279)	(0.2884)	(0.0032)	(0.4192)	(0.3518)
0.1	0.0283	0.0881	0.6944	0.8993	0.3039	0.1190
	(0.0160)	(0.0223)	(0.2705)	(0.0039)	(0.4110)	(0.2934)
0.2	0.0267	0.0846	0.5507	0.8074	0.2664	0.0868
	(0.0181)	(0.0194)	(0.2008)	(0.0057)	(0.3535)	(0.2413)
0.3	0.0281	0.0727	0.4791	0.7201	0.1698	0.1192
	(0.0149)	(0.0112)	(0.2202)	(0.0048)	(0.2760)	(0.2567)

### Application to real data

We apply scFSNN and several other classifiers to eight scRNA-seq datasets generated by different experimental protocols (Drop-seq, Smart-Seq2, CEL-Seq, inDrop and 10x-genomics). An overview of these datasets is given in Table 3. We filter out genes that have zero counts in more than $80\%$ of cells. The numbers of remaining genes are shown in Table 3. Here, we use the last name of the publication’s first author to denote each dataset. Adam [27] applied the cold protease scRNA-seq procedure to the newborn postnatal day 1 (P1) mouse kidney and clustered the isolated cells into nine classes(Cap Mesenchyme, Distal Tubule, Endothelial, Loop of Henle, Nephron Progenitor, Podocytes, Proximal Tubule, Stromal and Ureteric Bud). We use all nine cell types with a sample size of 4853 in the dataset. Dong [28] conducted scRNA-seq analysis of 1916 individual cells from eight organs and tissues of E9.5 to E11.5 mouse embryos. Here, we select 332 liver cells with three classes(E9.5, E10.5, E11.5) in our dataset. Bacher [29] investigated the low-avidity CD4+ T cell responses to SARS-CoV-2 in both unexposed individuals and patients with COVID-19. The cells, derived from 6 unexposed individuals and 14 COVID-19 patients, were classified as healthy, non-hospitalized, mild-moderate, or severe based on the donor’s health status and disease severity. We randomly selected 15,957 cells from these four categories for our classification task. Enge [30] contains 2282 pancreas cells from eight donors spanning six decades of life and comprising six categories: A cells, acinar cells, D cells, B cells, duct cells, and stellate cells. To identify rare cell types, Grun [31] sequenced the transcriptome of hundreds of randomly selected cells from mouse intestinal organoids. We use the count data from 1547 cells across 3 classes, including Reg4-positive cells, YFP-positive cells and Lgr5-positive cell, for classification analysis. Baron [32] unveiled the pancreas population structure with the transcriptomes of over 12000 pancreatic cells from four human donors and two mice. The dataset Baron includes all major cell groups from the human donors, excluding those with less than 200 cells. The cell types in Baron are acinar, activated stellate, alpha, beta, delta, ductal, endothelial and gamma. Chen [33] profiled transcriptomes of more than 14000 single cells and identified 45 transcriptionally distinct cell subtypes in the adult mouse hypothalamus. Based on the expression of the pan neuronal makers Snap25 and Syt1, the 45 cell clusters were divided into 34 neuronal(Snap25/Syt1-high) and 11 non-neuronal clusters(Snap25/Syt1-negative or low). We selected 7930 cells with 6 clusters (Astro, Tany, MO, OPC, Micro and Macro) out of the 11 non-neuronal clusters for the classification task. Alzheimer’s disease (AD) is the most common form of dementia but has no effective treatment. Lau [34] performed single-nucleus transcriptome analysis on 179392 nuclei from prefrontal cortical samples of twelve Alzheimer’s disease (AD) patients and nine normal control (NC) subjects. The data are categorized into AD group and NC groups based on disease status. For this dataset, we filtered out genes with zero counts in over $90\%$ of cells, resulting in 6569 genes for analysis. Due to the large dataset size, data augmentation was not utilized.

**Table 3:** Overview of datasets

Dataset	No. of samples	No. of genes	Platform	No. of cell types	References
Adam	4853	2710	Drop-seq	9	[27]
Dong	332	9627	Smart-Seq2	3	[28]
Bacher	15957	1944	10x-genomics	4	[29]
Enge	2282	6117	Smart-Seq2	6	[30]
Grun	1547	2821	CEL-Seq	3	[31]
Baron	8278	2988	inDrop	8	[32]
Chen	7930	1629	Drop-seq	6	[33]
Lau	179392	6569	Drop-seq	2	[34]

To assess the predictive performance of scFSNN and other classifiers, we employ a two-step procedure for splitting the dataset into training, validation, and test subsets. We first split the dataset into two non-overlapping portions: a training set for model training ($80\%$) and a test set ($20\%$) for performance evaluation. For methods that don’t require a validation set, we directly train the model on the training set and assess its accuracy on the test set. For methods like ours that require a validation set, we further split the training set into separate training and validation sets using an 8:2 ratio. The training set, containing $64\%$ of the cells, is used to update the deep learning model’s parameters, while the validation set,containing $16\%$ of the cells, help calculate feature importance scores and estimate the false discovery rate (FDR). The random split is repeated 20 times for each dataset, and average test accuracies are reported.

We compared scFSNN with nine other classifiers, including SurvNet, four variants of penalized methods, two generic classifiers and two classifiers specifically designed for RNA-seq dataset. The two generic classifiers are RF (Random Forest) and All-Feature (deep learning algorithm using all features in the datasets as input). In All-Feature network, the number of nodes in input layer is the number of genes and there are 256 and 128 nodes in two hidden layers, respectively. Batch normalization [20] and dropout [21] with a rate of 0.5 are used to accelerate deep network training and control overfitting. The loss function is cross entropy and Adam optimizer [35] with learning rate 0.001 is used to train the model.

The two classifiers designed for RNA-seq data are ZIPLDA [10] and SINC [7]. ZIPLDA is based on zero-inflated Poisson distribution and designed for bulk RNA-seq datasets. ZIPLDA ranks genes by the ratio of sum of squares between groups to within groups for each gene and selects first *K* genes. Here, we set $K=1000$ as recommended [10]. SINC is also a deep learning based algorithm. In data-preprocessing, SINC conducts an F-test on each gene to test whether different classes have significantly different mean expression levels and selects the top 1500 genes with smallest $P-$values as the input for deep learning neural network.

**Fig. 2:**
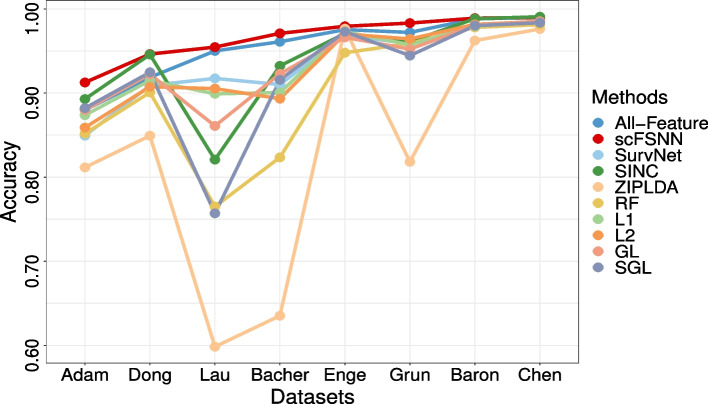
Predictive accuracies of scFSNN and nine other classifiers on eight scRNA-seq datasets. Results of different classifiers are shown in different colors

The results are summarized in Fig. 2 and Supplementary Tables S3 and S4. Overall, we find that scFSNN gives the highest predictive accuracies on seven out of the eight datasets, and its predictive accuracies are very close to the best on the other dataset (0.9897 versus 0.9907 for SINC on Chen). We also note that both SINC and All-Feature perform quite well in terms of predictive accuracy, and these two methods consistently outperform non-deep learning based methods RF and ZIPLDA. This indicates that deep learning can capture more complicated relationship between input and target than other generic and model-based classifiers.

**Fig. 3:**
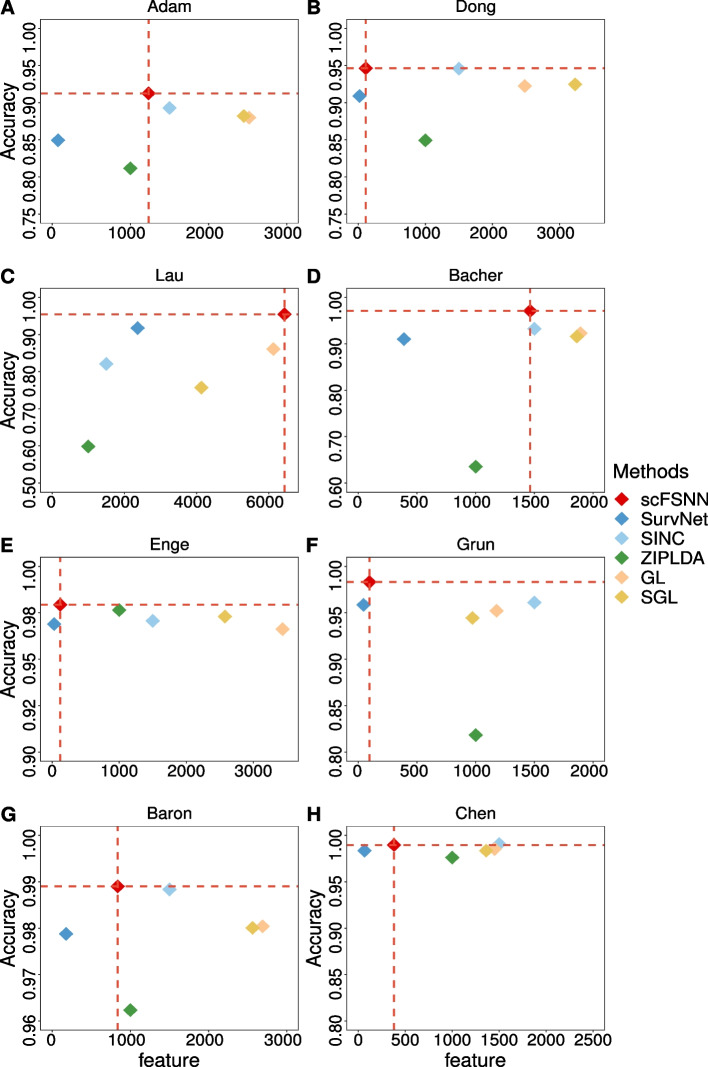
The number of selected features and predictive accuracy of scFSNN and five other classifiers with feature selection procedure on eight scRNA-seq datasets. **A** Adam. **B** Dong. **C** Lau. **D** Bacher. **E** Enge. **F** Grun. **G** Baron. **H** Chen. The number of selected features and predictive accuracy of scFSNN are shown as red dashed vertical and horizontal lines, respectively

To evaluate the sparsity of the model input, we also consider the number of selected features for scFSNN and five other classifiers which include feature selection in the procedures. In real datasets, we can not assess how many selected features are truly relevant to the target. In classification, our primary goals are twofold: first, to build a highly accurate model for predicting future observations, and second, to unveil the underlying relationships between features and the response variable, enriching our scientific understanding. In deep learning based methods, we do not expect to gain a concise relationship between each feature and the target. However, it is important to know which features are truly used in the model to make predictions. Therefore, we expect the final model to achieve high predictive accuracy with a small input size.

Figure 3, Supplementary Tables S3 and S5 show the number of selected features and predictive accuracy for six methods on the eight datasets. Note that we can not adaptively determine the number of features for SINC and ZIPLDA and fix them as 1500 and 1000, respectively. We can see that SINC achieves comparable predictive accuracy as scFSNN in many datasets, and it commonly uses many more features, except for Lau. SINC’s predictive accuracy is $82.3\%$ for the Lau dataset, indicating that it uses too few genes in the model. Notably, SurvNet selects smallest number of features in all datasets, but its predictive accuracy is also much smaller compared to scFSNN. This indicates that SurvNet may be too conservative and miss some important features. For the penalized methods, GL and SGL, they select a larger number of features than scFSNN, but the predictive accuracies are significantly smaller in all datasets.

To evaluate performance of scFSNN in terms of marker gene selection in a real dataset, we use the subdata of Baron dataset including cells from three healthy human donors with six types of cells (alpha, beta, gamma, delta, acinar and ductal). Baron dataset provides a list of 62 known marker genes for related cell types in pancreatic islets [32]. After filtering out genes with zero counts in more than $80\%$ cells, there are 50 marker genes remain. Both scFSNN and SINC achieve the highest predictive accuracy (98.86%), but scFSNN selects more marker genes (42 vs 25) while retaining fewer features (624 vs 1500) than SINC (Table 4). SurvNet has a slightly smaller predictive accuracy with 26 selected marker genes out of 134 genes in the model.

**Table 4:** Predictive accuracy, the number of selected features and marker genes of scFSNN and five other classifiers with feature selection procedure on Human datasets

Methods	Accuracy	Selected feature	Marker
scFSNN	0.9886	624	42
SurvNet	0.9837	134	26
SINC	0.9886	1500	25
ZIPLDA	0.9732	1000	8
GL	0.9827	2447	42
SGL	0.9818	2247	41

### Effects of $p_0$ and data augmentation

When estimating FDR, it is a common strategy to set $p_0$ as *p* for simplicity [11]. When the number of truly relevant features is small, which means $p_0$ is close to *p*, this strategy is reasonable. However, the number of truly relevant features may be non-negligible in scRNA-seq datasets. When this happens, $\hat{\eta }$ is overestimated and the procedure is too conservative. To assess the impact of estimates of $p_0$, we compare the proposed scFSNN with one that sets $p_0$ as *p*. We perform the feature selection procedures 50 times for each case and report the predictive accuracies.

**Fig. 4:**
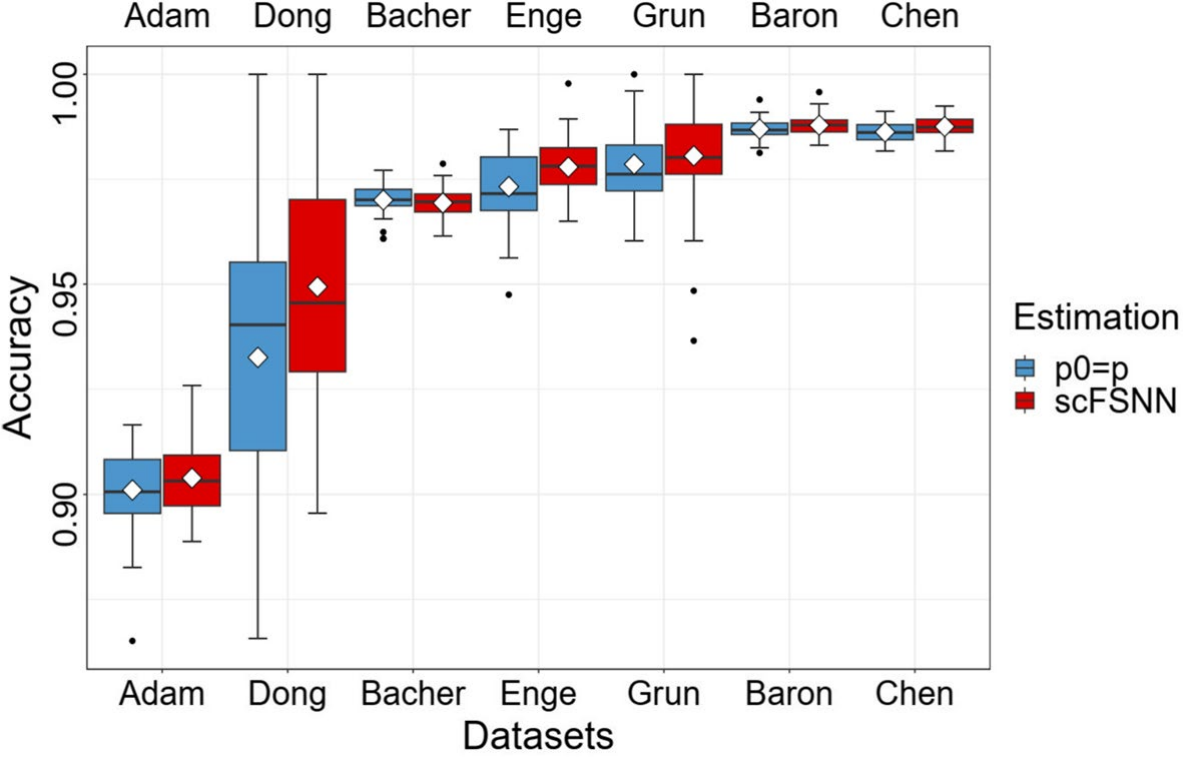
Predictive accuracies of the proposed scFSNN and scFSNN with $p_0=p$ on seven scRNA-seq datasets. The box represents the interquartile range, the horizontal line in the box is the median, the rhombus represent the average, and the whiskers represent the 1.5 times interquartile range

Data augmentation is a technique that artificially creates new training data from existing training data and is commonly used by DNN in computer vision. To assess its impact on scFSNN’s performance, we compared the proposed scFSNN with a version without data augmentation. This experiment was repeated 50 times.

**Fig. 5:**
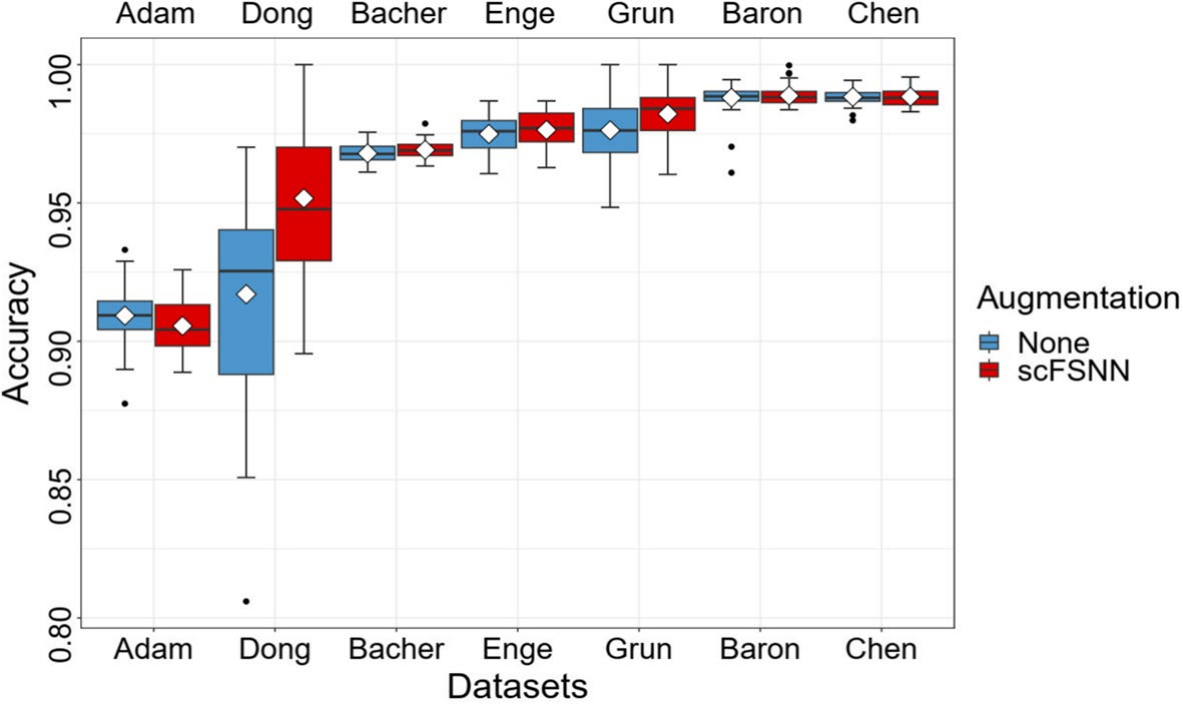
Predictive accuracies of the proposed scFSNN and scFSNN without data augmentation on seven scRNA-seq datasets. The box represents the interquartile range, the horizontal line in the box is the median, the rhombus represent the average, and the whiskers represent the 1.5 times interquartile range

Figure 4 and Supplementary Table S6 show that setting the hyperparameter $p_0$ to $\textrm{min}(\#\{S_j<S_m\}\times 2, p)$ leads to statistically significant improvements in accuracy for three datasets and comparable accuracy in others for scFSNN. Furthermore, Fig. 5 and Supplementary Table S7 reveal that data augmentation significantly enhances performance on the two small-scale datasets, Dong and Grun. For the remaining dataset, scFSNN again exhibits comparable predictive accuracy.

For real-world applications of scFSNN, we recommend considering both $p_0$ estimation and data augmentation as they have the potential to improve model performance.

### Influence of the normalization method

We utilized the total counts normalization method (TCN), wherein each cell’s counts are divided by its total counts and then multiplied by a scale factor (we set the scale factor as the median total counts across all cells). The resulting values are then natural-log transformed using log1p. This approach bears resemblance to Seurat’s log normalization method [36] and is a widely adopted, straightforward option for normalizing scRNA-seq data. To stabilize the training process, we further standardized the normalized data, making its mean and standard deviation become 0 and 1, respectively.

To illustrate the influence of the normalization method throughout the procedure, we assessed the performance of feature selection methods across three datasets, employing three different normalization methods: TCN, TMM [37], and SCTransform [38]. As shown in the Table 5, all three normalization methods perform similarly on all datasets.

**Table 5:** Predictive accuracies of the scFSNN with 3 normalization methods, TCN, TMM and SCTransform on 4 scRNA-seq datasets

Dataset	TCN	TMM	SCTransform
Grun	0.9822	0.9835	0.9805
	(0.0070)	(0.0083)	(0.0066)
Baron	0.9888	0.9889	0.9889
	(0.0031)	(0.0024)	(0.0024)
Chen	0.9884	0.9871	0.9894
	(0.0033)	(0.0028)	(0.0028)
Lau	0.9546	0.9556	0.9539
	(0.0020)	(0.0030)	(0.0038)

## Conclusions

Fast and accurate feature selection is critical for large-scale classification analysis in scRNA-seq datasets. Finding informative gene sets from numerous candidates can greatly enhance explanatory ability, improve predictive accuracy, and reduce the labor and cost of applying scRNA-seq to clinical tests, therapeutic discovery and genetic screens. In this paper, we have proposed an embedded algorithm for the classification of samples based on the DNN framework, scFSNN, that incorporate a fast and simple backward feature selection procedure. scFSNN can adaptively choose the number of genes to be deleted at each step, thus accelerating the feature selection procedure. scFSNN controls the FDR by generating a set of null genes to estimate the null distribution, avoiding the use of methods that have highly computational burden, such as cross-validation. Furthermore, by using data augmentation, scFSNN can achieve high generalization predictive abilities. Our experimental results demonstrate that our scFSNN algorithm achieve higher predictive accuracy with substantially informative genes than other algorithms for scRNA-seq datasets. Based on these results, we believe that scFSNN can be fruitfully applied to many scRNA-seq datasets.

### Supplementary Information

**Supplementary material 1.**

## Data Availability

The code developed for the study of scFSNN is publicly available at the Github repository https://github.com/linbingqing/scFSNN. All scRNA-seq data used in this paper are available publicly in Gene Expression Omnibus under accession number GSE94333 (Adam [27]), GSE87038 (Dong [28]), GSE62270 (Grun [31]), GSE81547 (Enge [30]), GSE84133 (Baron [32]) and GSE87544 (Chen [33]), GSE157827 (Lau [34]).
