# Supplementary material for: scFSNN: a feature selection method based on neural network for single-cell RNA-seq data
Source: BMC Genomics. 2024 Mar 8;25:264. doi: 10.1186/s12864-024-10160-1 (PMC10924397; doi:10.1186/s12864-024-10160-1)
Supplement: Supplementary file 1 — Supplementary material 1. [file 12864_2024_10160_MOESM1_ESM.pdf]

scFSNN: a feature selection method based on neural network for single-cell RNA-seq data
Supplementary material

Minjiao Peng [1,2], Baoqin Lin[3], Jun Zhang[1],
Yan Zhou[1], Bingqing Lin [1*]
[1]School of Mathematical Sciences, Shenzhen University,
Shenzhen 518060, China.
[2]School of Mathematics and Statistics and KLAS, Northeast Normal University,
Changchun 130000, China.
[3]Experimental Center,
The First Affiliated Hospital of Guangzhou University of Chinese Medicine,
Guangzhou 510405, China

1 Supplementary Tables

Table S1: Predictive accuracy comparison of scFSNN and five other classifiers on five simulated scRNA-seq datasets with varying sample sizes.

sample size	scFSNN	SurvNet	L_1	L_2	GL	SGL
1000	1	1	1	1	0.9739	0.9604
2000	1	0.9999	0.9351	0.9999	0.9240	0.9741
3000	1	1	1	0.9743	0.9634	1
4000	1	1	0.9245	0.9999	0.9758	1
5000	1	0.9998	1	1	0.9999	0.9999

Table S2: Predictive accuracy comparison of scFSNN and five other classifiers on five simulated scRNA-seq datasets with varying proportions of DE genes.

DE	scFSNN	SurvNet	L_1	L_2	GL	SGL
0.02	0.9997	0.9996	0.9255	0.9781	0.9728	0.9744
0.03	1	0.9999	1	0.9998	0.9604	0.9746
0.05	1	1	1	0.9542	0.9751	0.9966
0.1	1	1	0.9684	1	0.9838	1
0.2	1	1	1	1	0.9510	0.9762
0.3	1	1	1	1	1	1

Table S3: Predictive accuracies of scFSNN and nine other classifiers on eight scRNA-seq datasets. Bold black values represent the highest accuracy achieved by any method for the corresponding dataset. Standard errors are shown in parentheses.

Dataset	All-Feature	scFSNN	SurvNet	SINC	RF	ZIPLDA	L_1	L_2	GL	SGL
Adam	0.8799	**0.9125**	0.8493	0.8928	0.8517	0.8115	0.8736	0.8589	0.8798	0.8820
	(0.0083)	(0.0092)	(0.0285)	(0.0243)	(0.0031)	(0.0118)	(0.0097)	(0.0156)	(0.0113)	(0.0125)
Dong	0.9187	**0.9463**	0.9090	0.9458	0.9007	0.8493	0.9149	0.9075	0.9224	0.9246
	(0.03)	(0.0288)	(0.0639)	(0.0475)	(0.0147)	(0.0359)	(0.0339)	(0.0407)	(0.0268)	(0.0334)
Lau	0.9500	**0.9546**	0.9174	0.8208	0.7650	0.5984	0.8990	0.9052	0.8609	0.7570
	(0.001)	(0.002)	(0.005)	(0.0111)	(0.0023)	(0.0059)	(0.0139)	(0.0049)	(0.0169)	(0.1556)
Bacher	0.9609	**0.9709**	0.9101	0.9323	0.8235	0.6353	0.9006	0.8933	0.9228	0.9157
	(0.0034)	(0.0029)	(0.008)	(0.011)	(0.0048)	(0.0068)	(0.0096)	(0.0055)	(0.0161)	(0.008)
Enge	0.9754	**0.9793**	0.9689	0.9707	0.9479	0.9765	0.9730	0.9701	0.9662	0.9730
	(0.0081)	(0.0048)	(0.0089)	(0.0143)	(0.0036)	(0.0064)	(0.0067)	(0.0069)	(0.0243)	(0.0091)
Grun	0.9720	**0.9831**	0.9585	0.9611	0.9589	0.8183	0.9573	0.9643	0.9522	0.9444
	(0.012)	(0.0073)	(0.0214)	(0.0278)	(0.0065)	(0.0229)	(0.0166)	(0.0143)	(0.0203)	(0.0186)
Baron	0.9882	**0.9890**	0.9788	0.9883	0.9780	0.9623	0.9794	0.9816	0.9804	0.9801
	(0.0017)	(0.0031)	(0.0045)	(0.006)	(0.0022)	(0.0044)	(0.0056)	(0.0037)	(0.0038)	(0.0036)
Chen	0.9898	0.9897	0.9835	**0.9907**	0.9812	0.9761	0.9851	0.9851	0.9853	0.9835
	(0.0022)	(0.0024)	(0.0037)	(0.0042)	(0.0014)	(0.0041)	(0.0033)	(0.0034)	(0.0034)	(0.0034)

Table S4: The t-test results comparing means of classification accuracies between scFSNN and nine other classifiers across eight scRNA-seq datasets. An asterisk (*) indicates a p-value below 0.05, signifying that scFSNN outperforms the corresponding method. Hyphens (-) represent values greater than or equal to 0.05.

Dataset	All-Feature	SurvNet	SINC	RF	ZIPLDA	L_1	L_2	GL	SGL
Adam	*	*	*	*	*	*	*	*	*
Dong	*	*	-	*	*	*	*	*	*
Lau	*	*	*	*	*	*	*	*	*
Bacher	*	*	*	*	*	*	*	*	*
Enge	-	*	*	*	-	*	*	*	*
Grun	*	*	*	*	*	*	*	*	*
Baron	-	*	-	*	*	*	*	*	*
Chen	-	*	-	*	*	*	*	*	*

Table S5: Number of selected features of scFSNN and five other classifiers with feature selection procedures on eight scRNA-seq datasets.

Dataset	scFSNN	SurvNet	SINC	ZIPLDA	GL	SGL
Adam	1232	76	1500	1000	2517	2450
Dong	111	18	1500	1000	2481	3232
Lau	6433	2368	1500	1000	6126	4133
Bacher	1464	389	1500	1000	1893	1862
Enge	121	30	1500	1000	3436	2576
Grun	94	44	1500	1000	1178	972
Baron	838	178	1500	1000	2690	2562
Chen	380	66	1500	1000	1451	1361

Table S6: Predictive accuracies and corresponding standard deviations achieved by scFSNN and scFSNN with $p_0 = p$. An asterisk (*) indicates a p-value below 0.05, indicating that the scFSNN significantly outperforms the other one.

Dataset	scFSNN	scFSNN with $p_0 = p$	Dataset	scFSNN	scFSNN with $p_0 = p$
Adam	0.9039	0.9010	Grun	0.9806	0.9786
	(0.0097)	(0.0103)		(0.0122)	(0.0077)
Dong	0.9494*	0.9325	Baron	0.9879	0.9870
	(0.0255)	(0.0326)		(0.0023)	(0.0022)
Bacher	0.9693	0.9700	Chen	0.9876*	0.9863
	(0.0035)	(0.0035)		(0.0024)	(0.0023)
Enge	0.9779*	0.9732			
	(0.0062)	(0.0083)			

Table S7: Predictive accuracies and corresponding standard deviations achieved by scFSNN and scFSNN without data augmentation. An asterisk (*) indicates a p-value below 0.05, indicating that the scFSNN significantly outperforms the other one.

Dataset	scFSNN	scFSNN without data augmentation	Dataset	scFSNN	scFSNN without data augmentation
Adam	0.9055	0.9092	Grun	0.9822*	0.9763
	(0.0095)	(0.0105)		(0.0079)	(0.0107)
Dong	0.9517*	0.9170	Baron	0.9888	0.9880
	(0.0286)	(0.0375)		(0.0036)	(0.0054)
Bacher	0.9692	0.9679	Chen	0.9884	0.9883
	(0.0030)	(0.0034)		(0.0032)	(0.0029)
Enge	0.9764	0.9749			
	(0.0058)	(0.0065)			

2 Supplementary Figures

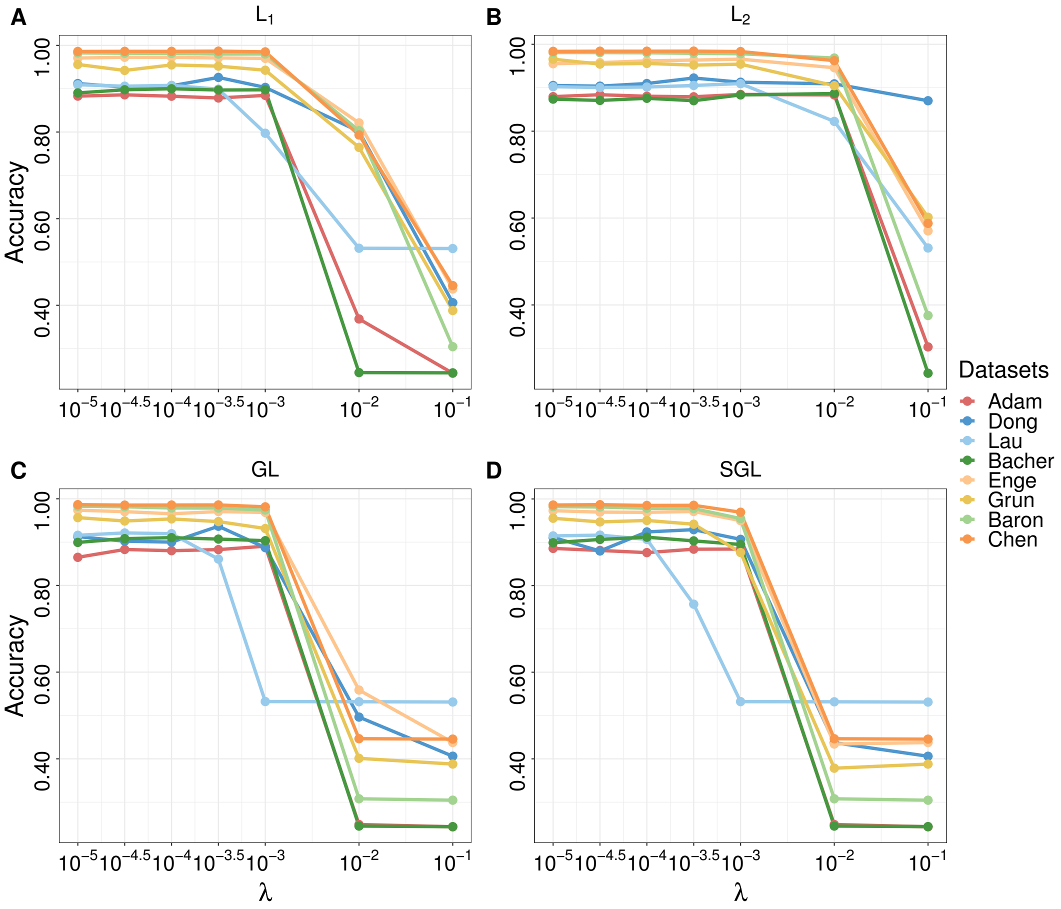

Figure S1: The predictive accuracies of four penalized methods (A: L_1, B: L_2, C: GL, D: SGL) are shown for eight scRNA-seq datasets with varying tuning parameters. Different colors represent results for different datasets. Overall, the accuracies of all four methods decrease with the increase in tuning parameters.

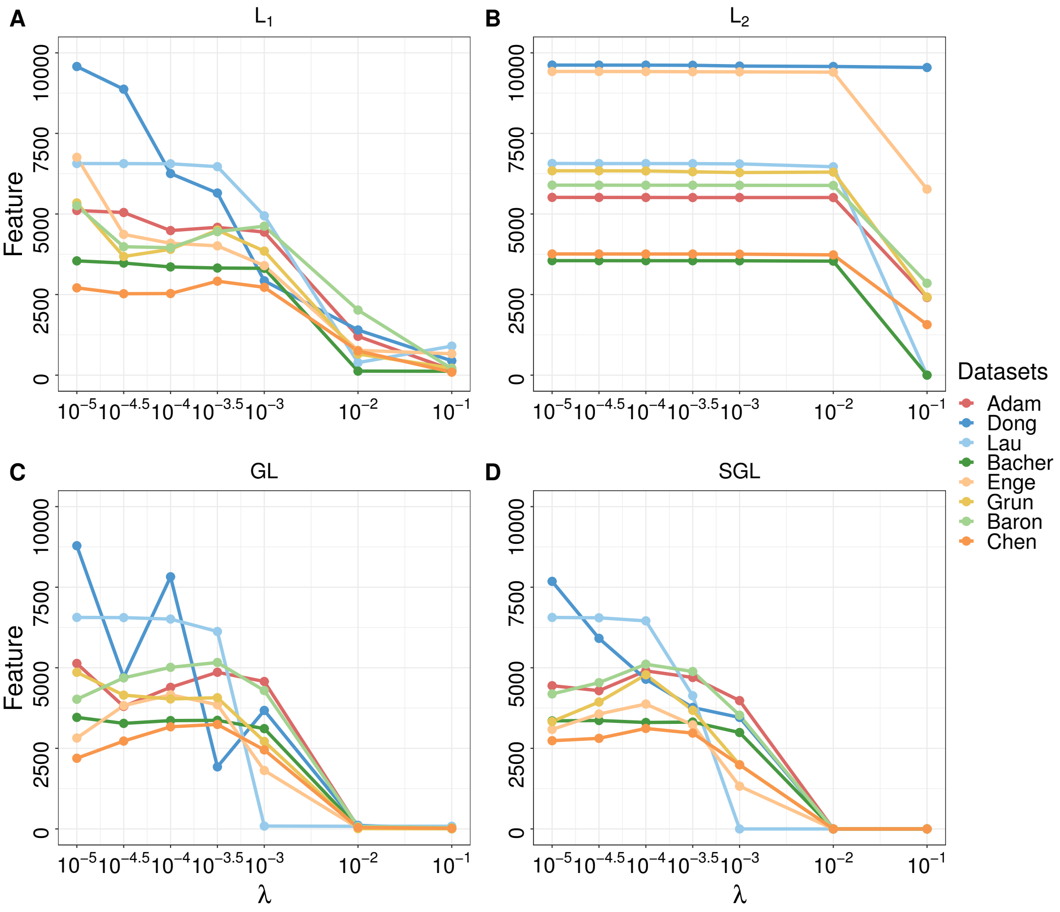

Figure S2: The number of selected features of four penalized methods(A: L_1, B: L_2, C: GL, D: SGL) are shown for eight scRNA-seq datasets with different tuning parameters. Results for different datasets are represented by distinct colors. Overall, the number of selected features of all four methods decreases as the tuning parameters increase.
